# Efficacy of Phlai (*Zingiber montanum*) Spray Cool Formula in Managing Upper Trapezius Myofascial Pain Syndrome: A Randomized Controlled Trial

**DOI:** 10.3390/life15030360

**Published:** 2025-02-25

**Authors:** Prakairat Tunit, Nurmee Mahama, Nursawiyah Mina, Nasrin Chi, Suwanna Maenpuen, Pornchai Sawangwong, Waratta Hemtong, Phasit Sirited, Chuda Chittasupho

**Affiliations:** 1 Thai Traditional Medicine Program, Faculty of Nursing and Allied Health Sciences, Phetchaburi Rajabhat University, Phetchaburi 76000, Thailand; prakairat.tun@mail.pbru.ac.th (P.T.); nurmee7783@gmail.com (N.M.); nursawiyamina@gmail.com (N.M.); nasrin.chi@mail.pbru.ac.th (N.C.); waratta.hem@mail.pbru.ac.th (W.H.); 2 Applied Thai Traditional Medicine Program, Faculty of Medicine, Mahasarakham University, Mahasarakham 44000, Thailand; suwanna.m@msu.ac.th; 3 Thai Traditional and Integrated Medicine Hospital, Department of Thai Traditional and Alternative Medicine, Ministry of Public Health, Nonthaburi 11000, Thailand; pornchai093@gmail.com; 4 Public Health Program, Faculty of Nursing and Allied Health Sciences, Phetchaburi Rajabhat University, Phetchaburi 76000, Thailand; phasit2530@gmail.com; 5 Department of Pharmaceutical Sciences, Faculty of Pharmacy, Chiang Mai University, Chiang Mai 50200, Thailand

**Keywords:** myofascial pain syndrome, clinical trial, stability, curcumin, β-sitosterol

## Abstract

Phlai (*Zingiber montanum*) has long been valued for its anti-inflammatory and analgesic properties in traditional medicine. This study aimed to develop and assess the physical stability, chemical composition, and clinical efficacy of a novel Phlai spray cool formula (PSCF) compared to a diclofenac spray (DS) in patients with chronic myofascial pain syndrome. The chemical analysis revealed curcumin (28.73 ± 5.73 mg/100 g), β-sitosterol (50.92 ± 1.27 mg/100 g), and lauric acid (38.86 ± 1.72 g/100 g) as key active compounds. PSCF demonstrated stable physicochemical properties, including pH and peroxide value across storage conditions. In a randomized controlled trial involving 66 participants, PSCF and DS groups exhibited comparable reductions in pain intensity, as measured by the Visual Analog Scale (VAS), from baseline to week 2. Both groups also showed significant improvements in neck disability index (NDI), pressure pain threshold (PPT), and cervical range of motion (CROM). By week 2, the increase in CROM for flexion and extension reached 23.54 ± 4.09° and 19.43 ± 3.20°, respectively, with no significant intergroup differences. The SF-36 health survey indicated notable improvements in overall health status and quality of life, particularly in physical and emotional domains. The analgesic effects of PSCF are attributed to the combined action of menthol, β-sitosterol, and curcumin. The study demonstrated that PSCF offers a therapeutic effect comparable to diclofenac spray without adverse reactions, highlighting its potential as an alternative topical analgesic for chronic myofascial pain management.

## 1. Introduction

Myofascial pain syndrome (MPS) is a soft tissue disorder characterized by localized and referred musculoskeletal pain originating from trigger points. It is one of the most prevalent causes of both acute and chronic pain [1]. Estimates suggest that 20% to 95% of patients with musculoskeletal pain seen by general physicians or pain specialists may have myofascial pain. Several studies report a prevalence of up to 85% in the general population, with varying gender distribution, though some evidence indicates higher rates in females [2]. Approximately 85% of patients attending chronic pain clinics and 30% visiting internal medicine clinics have been diagnosed with myofascial pain [3]. Notably, it accounts for 85% of back pain and over half of chronic head and neck pain [4]. In a study of patients with non-specific neck pain, 100% were diagnosed with myofascial pain syndrome [3]. These findings underscore the significance of recognizing and managing myofascial pain, as it is a major contributor to chronic regional pain, including chronic back, shoulder, head, and facial pain. Myofascial pain syndrome, defined by the presence of pain originating from skeletal muscle trigger points, is a frequent component of regional musculoskeletal pain disorders, occurring either in isolation or combined with other pain generators [5]. The development of trigger points (TrPs) typically occurs secondary to muscular trauma or microtrauma, necessitating a thorough investigation of activities, events, or behavioral patterns that could lead to muscular overload, excessive stretching, direct injury, repetitive stress, overuse, or disuse [6]. Evidence suggests that the principal long-term effects of neck pain manifest as individual functional disability and occupational absenteeism, both recognized as significant challenges to public health and socioeconomic stability [7,8].

Active trigger points in the upper trapezius exhibit a distinct biochemical profile, including elevated levels of inflammatory mediators and nociceptive substances such as neuropeptides, cytokines, and bradykinin. This unique molecular signature, comprising elevated concentrations of protons, neuropeptides (SP, CGRP), bradykinin, pro-inflammatory cytokines (TNF-α, IL-1, IL-6, IL-8), 5-HT, and norepinephrine, differed significantly from measurements obtained from the control site in the medial gastrocnemius muscle [9]. The pharmacological management of myofascial pain encompasses several drug classes, including analgesics, muscle relaxants, anticonvulsants, and antidepressants [5]. Various non-pharmacological treatment options have been investigated, ranging from acupuncture and traditional Thai massage to muscle stretching and meditation [10,11] However, each therapeutic approach has specific benefits and limitations that warrant careful evaluation. Particularly severe cases with complicated presentations typically require a comprehensive treatment strategy combining multiple therapeutic modalities [10].

Phlai (*Zingiber montanum*) from the Zingiberaceae family is an herbaceous plant that originated in Southeast Asia [12]. The mature rhizomes of Phlai are considered its most valuable and medicinally significant part, although all parts of the plant have practical applications in traditional medicine. The pharmacological properties of this plant include anti-inflammatory, antifungal, and anti-bacterial activities [13,14]. Furthermore, Phlai has demonstrated therapeutic properties for musculoskeletal conditions, specifically in muscle recovery and alleviating muscular and joint pain [15]. Chemical analysis of the rhizome revealed various bioactive compounds, including alkaloids, saponins, tannins, flavonoids, terpenoids, and phenolic compounds [16]. The traditional coconut oil extraction of Phlai has been officially endorsed in Thailand’s healthcare system since 2011, when it was included in the National List of Essential Medicines.

A comprehensive review of Phlai’s use in managing musculoskeletal pain identified three primary clinical categories: upper back disorders, including temporomandibular joint dysfunction and cervicobrachial pain [17,18]; occupational muscle strain, supported by four pivotal studies [19,20,21,22]; and chronic myofascial pain syndrome [23,24]. Clinical trials comparing Phlai-containing herbal compress therapy with topical diclofenac gel demonstrated notable therapeutic advantages in managing myofascial pain syndrome, with significant improvements in pain reduction, cervical range of motion (CROM), pressure pain threshold (PPT), and overall quality of life [23]. While Phlai oil (1:3 ratio in palm oil, 91.5% concentration) demonstrated analgesic efficacy comparable to diclofenac gel in chronic muscle pain management, the observation of similar pain-relieving effects in the methyl salicylate placebo group necessitates further phytochemical investigation to elucidate the specific active compounds responsible for Phlai oil’s therapeutic action [24]. Despite these promising findings, the chemical composition of coconut oil-extracted Phlai oil and its formulation as a cooling topical spray remain underexplored. This study addresses this gap by analyzing the chemical constituents and physical stability of Phlai spray cool formula. Additionally, its therapeutic efficacy will be evaluated against diclofenac spray in patients with MPS, focusing on pain reduction, range of motion, neck disability, and overall health status.

## 2. Materials

Rhizomes of *Zingiber montanum* aged one year were obtained from the local market in Nong Ya Plong District, Phetchaburi Province, Thailand. Coconut oil was obtained from Chemipan Corporation Co., Ltd. (Bangkok, Thailand). Menthol, borneol, and camphor were purchased from Krungthepchemi Co., Ltd. (Bangkok, Thailand).

## 3. Methods

### 3.1. PSCF Preparation

The preparation process involved finely chopping fresh Phlai rhizomes and frying them with coconut oil (2:1 *w*/*w* ratio) for approximately one hour until achieving a brownish-crispy consistency. The resulting yellowish oil was paper-filtered and stored under dark, cool conditions. The Phlai spray cool formula (PSCF) was prepared by mixing menthol (14% *w*/*w*), camphor (10% *w*/*w*), and borneol (4.46% *w*/*w*). Then, the mixed solution was added to the Phlai oil (53% *w*/*w*). The completed mixture was stored in amber spray bottles.

### 3.2. Determination of Curcumin in PSCF

The curcumin content in PSCF was quantified using High-Performance Liquid Chromatography (HPLC) with Diode Array Detection (LC-DAD). The analysis followed the in-house method TE-CH-419, which is based on the methodology outlined in the *Journal of AOAC International*, Vol. 101, No. 1, 2018, pp. 203–207, employing the LC-DAD technique.

### 3.3. Determination of the Fatty Acid Composition in PSCF

The fatty acid composition in PSCF was analyzed using the Compendium of Methods for Food Analysis following the in-house method WL-TMC-05, based on AOAC (2023) methods 996.06 and 969.33. PSCF (2.0 g) was weighed into an Erlenmeyer flask and extracted with 50 mL of chloroform/methanol (2:1 *v*/*v*). The mixture was agitated vigorously for 30 min. The upper layer was then filtered through Whatman No. 1 filter paper and mixed with anhydrous sodium sulfate to adsorb excess water. This extraction process was repeated twice, and the combined filtrate was subjected to rotary evaporation to remove the organic solvent. The resulting residue was weighed and dissolved in 5 mL of 0.5 N potassium hydroxide in methanol. Tricosanoic acid methyl ester (800 µg/mL, 1 mL) was added as an internal standard in quantitative analysis.

The mixture was placed in a water bath shaker at 100 °C for 5 min. After the mixture was cooled, 14% borontrifluoride (BF3) in methanol (2 mL) was added. The mixture was then placed in a water bath shaker at 100 °C for 15 min. After adding saturated sodium chloride solution (10 mL) to the mixture, the supernatant was further extracted with petroleum ether (5 mL/time) until a clear solution was obtained. All extracts were collected and rotary-evaporated under controlled temperature and pressure until dry. The mixture was mixed with n-heptane (3 mL) and filtered through a nylon syringe filter 13 mm in diameter, 0.45 µm, before analysis using a 7890A Gas Chromatograph (Agilent, Santa Clara, CA, USA).

### 3.4. Quantification of Plant Sterols in PSCF by Gas Chromatography

Plant sterol quantification in PSCF was conducted using gas chromatography [25]. Stock solutions (500 ppm) of beta-sitosterol, stigmasterol, campesterol, and brassicasterol were prepared in n-hexane at a 0.5 mg/mL concentration. Working solutions (10–200 ppm) were obtained through the serial dilution of the stock solutions with n-hexane and stored at −20 °C. The derivatization process involved combining the plant sterol standards with trimethylsilyl (TMS) pyridine (9 mL), hexane (3 mL), and chloroform (1 mL), followed by the addition of a 100 µL sample. The resulting derivatives underwent nitrogen drying and filtration prior to chromatographic analysis.

Sample preparation involved the liquid extraction and saponification of PSCF (2.5 g) with 5-α-cholestan (50 ppm internal standard, 1 mL) and ethanolic potassium hydroxide (1 mL). The mixture was homogenized for 1 min and heated (60 °C, 30 min). The sequential addition of deionized water (5 mL) and chloroform (10 mL) was followed by 2 min of mixing. After collecting the upper layer, the extraction was repeated with hexane, and the combined upper layers were evaporated to dryness at 50 °C.

Plant sterol derivatization of the PSCF samples followed a previously described protocol [26]. Chromatographic analysis was performed using an Agilent 6850 Series Gas Chromatography System equipped with a methylsiloxane capillary column (HP1, 30 m × 0.32 mm i.d., 0.25 µm film thickness). The analytical parameters included an inlet temperature of 300 °C and a temperature program starting at 200 °C (1 min), increasing at 5 °C/min to 290 °C, with a carrier gas flow rate of 1 mL/min.

### 3.5. Peroxide Value of PSCF

The peroxide value of the PSCF was assessed using the iodometric titration method [25]. The procedure involved mixing 5 g of PSCF with 30 mL of an acetic acid–chloroform solution (3:2 *v*/*v*), followed by adding 1 mL of potassium iodide. After allowing the reaction to proceed for one minute, 30 mL of water and 0.5 mL of starch solution were added sequentially. The resulting mixture was then titrated with 0.01 N sodium thiosulfate until the endpoint was reached.

### 3.6. Color Measurement of PSCF

The colorimetric analysis of the PSCF was performed using a HunterLab spectrophotometer (HunterLab, Reston, VA, USA), measuring L* (lightness), a* (red/green), and b* (yellow/blue) coordinates [25].

### 3.7. Study Designs and Ethics

A double-blinded randomized controlled trial was conducted using a randomized complete block design (RCBD) based on age, shoulder pain severity, and range of motion.

To ensure concealed allocation, opaque sealed envelopes were utilized in the randomization process. For blinding purposes, the diclofenac spray containers were modified to match the yellow color of the PSCF, making them visually indistinguishable to maintain the double-blind design.

This study was conducted in accordance with the Declaration of Helsinki and received ethical approval from the Human Research Ethics Committee of Phetchaburi Rajabhat University (No. 7/2024). The study protocol was registered with the Thai Clinical Trials Registry (TCTR20241202001). Written informed consent was obtained from all participants prior to study enrollment.

### 3.8. Participants

**Figure 1:**
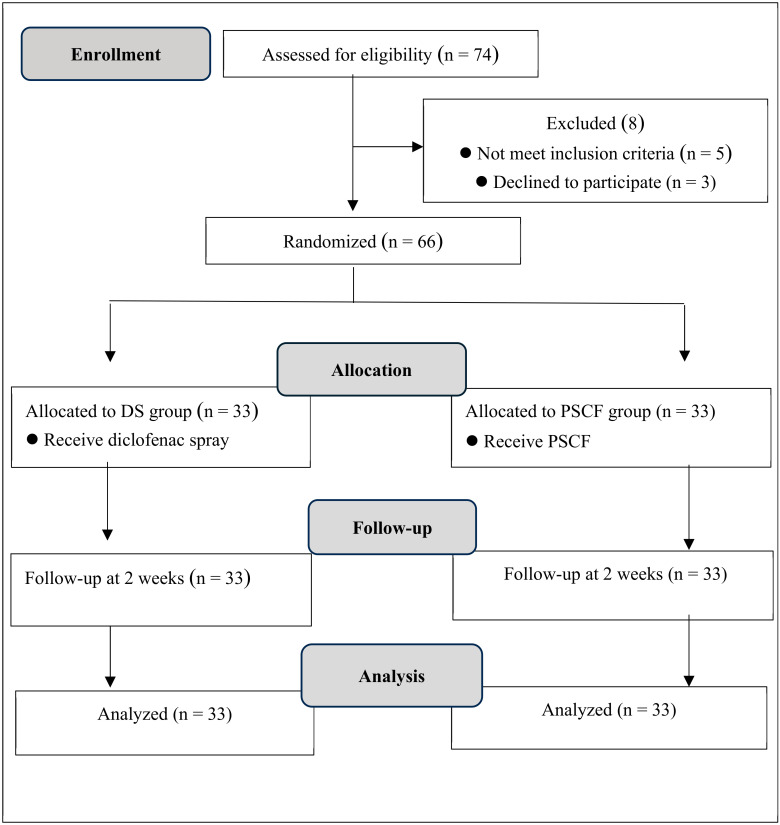
Study flowchart.

The sample size was calculated using G*Power 3.1.3 with a power of 0.8 and an alpha level of 0.05 [27]. The required sample size was 60 participants, which increased by 10% to 66 participants (33 per group) to account for potential dropouts. The study employed a double-blinded design comparing PSCF (experimental group) with DS (control group). Figure 1 shows the study flowchart. The study was conducted from April to September 2024.

### 3.9. Inclusion Criteria

1. Patients aged 18–60 who had suffered from myofascial pain syndrome in the upper trapezius for at least 3 months.
2. History of having used a computer continuously for 1 year.
3. Visual analog score of at least 3.
4. The upper trapezius exhibits muscular tightness in bands, along with pain referral patterns and trigger point indicators.
5. Had not used analgesic medication during the last month.
6. Consented to maintain participation for the complete study timeline.

### 3.10. Exclusion Criteria

1. Pregnancy.
2. History of hypersensitivity to Phlai oil, components in PSCF, or diclofenac preparations.
3. History of steroid or herbal medication intake during the 14-day period prior to study participation.
4. History of bone fractures or shoulder injury.

### 3.11. Study Intervention

The participants were required to complete three assessment tools: (1) the Visual Analog Scale (VAS) for pain intensity measurement, (2) the Neck Disability Index (NDI) for functional disability assessment, and (3) the 36-Item Short-Form Health Survey (SF-36) for health status evaluation. The research team conducted objective measurements using a goniometer for an ROM assessment and an algometer for pressure pain threshold evaluation. All measurements were documented in designated forms: (1) record form and (2) CROM assessment form. Then, the participants were instructed by the researcher on the application methods of PSCF and DS, accompanied by detailed explanations of data recording procedures in the research manual. The participants continuously applied 2 mL of the PSCF or DS three times daily (morning, afternoon, and evening) to the affected shoulder(s) for two weeks. As DS was a clear liquid, a yellow colorant was incorporated to achieve visual consistency with PSCF. Protocol compliance was monitored through the examination of product containers, which participants were required to present during weeks 1 and 2. Based on previous studies, a 2-week treatment period was selected as it has been shown to be sufficient time to observe topical therapeutic effects in myofascial pain syndrome treatment [28].

In week 1, the participants completed VAS and NDI questionnaires. ROM and measurements were conducted using a goniometer and algometer, respectively, with results documented in appropriate record forms.

In week 2, the participants completed VAS, NDI, and SF-36 questionnaires and returned their research manuals. The research team repeated the goniometer and algometer measurements, documenting the results in the ROM and PPT record forms.

## 4. Analytical Tools

### 4.1. Pain Intensity

The Visual Analog Scale (VAS) consists of a 10 cm continuous spectrum designed to quantify subjective characteristics or attitudes that are challenging to measure directly. The scale categorized pain levels as 0 (no pain), 1–2 (occasional), 3–4 (mild), 5 (moderate), 6–7 (severe), 8–9 (very severe), and 10 (intolerable requiring intervention) [29].

### 4.2. Neck Disability Index (NDI)

The Neck Disability Index (NDI) comprises 10 items with 6 options each, scored 0–50: 0–4 (no disability), 5–14 (mild), 15–24 (moderate), 25–34 (severe), and 35–50 (complete disability). The Thai version of the NDI is a valid and reliable measurement method for evaluating neck pain disability [30].

### 4.3. Pressure Pain Threshold (PPT)

Pressure pain threshold (PPT) was measured using a digital pressure algometer with a 1 cm^2^ probe in kilopascals (kPa). The participants sat in a standardized position: upright with their head in the neutral position, 90° elbow and knee flexion, feet flat, and back against chair support. A handheld switch let participants indicate when pressure (applied at 40 kPa/s) transitioned to initial pain sensation at the most sensitive upper trapezius trigger point. The instrument demonstrated validated reliability [31].

### 4.4. Cervical Range of Motion (ROM)

The cervical range of motion was assessed in paired movements for six positions: cervical flexion/extension, right/left lateral flexion (side bending), and right/left cervical rotation [32].

### 4.5. Health-Related Quality of Life Assessment

The SF-36 health survey measures quality of life across eight dimensions: physical functioning, physical role, social functioning, emotional role, bodily pain, mental health, vitality, and general health perception. Each dimension is scored from 0 (worst) to 100 (best health status).

### 4.6. Instrument Validation

The study’s assessment tools were validated by two Thai traditional medicine practitioners and one physiotherapist. Record forms for pain, PPT, and ROM measurements achieved an Item-Objective Congruence (IOC) score of 1.0, indicating perfect agreement. Questionnaires for the Neck Disability Index (NDI) and SF-36 health survey achieved a Content Validity Index (CVI) score of 0.9, demonstrating strong validity.

### 4.7. Statistical Analysis

Descriptive statistics for demographics (gender, age, weight, BMI, shoulder pain location) and within-group comparisons of VAS, NDI, SF-36, ROM, and PPT at baseline, week 1, and week 2 were assessed using repeated-measures ANOVA, followed by Bonferroni post hoc testing for pairwise comparisons where significant differences were detected. The normality of the data distribution was assessed using the Shapiro–Wilk test. Qualitative data were presented as proportions, and the chi-square test was used to test for significance. Within-group comparisons using quantitative data employed paired *t*-tests, while independent sample *t*-tests were used to compare between the two groups at each time point with statistical significance set at *p* < 0.05.

## 5. Results

### 5.1. Physical and Chemical Characterizations of PSCF

#### 5.1.1. Amount of Curcumin in PSCF

In the PSCF, curcumin content was identified with a retention time peak of 7.6 min. By calculating the area under the curve (AUC) and comparing it with a curcumin standard, the curcumin content in 100 g of PSCF was determined to be 28.73 ± 5.73 mg.

#### 5.1.2. Fatty Acid Composition in PSCF

The fatty acid composition analysis of 100 g of PSCF revealed the presence of both saturated and unsaturated fatty acids. The saturated fatty acids included lauric acid, myristic acid, palmitic acid, undecanoic acid, capric acid, stearic acid, caprylic acid, and arachidic acid. The unsaturated fatty acids identified were cis-9-oleic acid and linoleic acid. The amounts of fatty acid compositions are summarized in Table 1.

#### 5.1.3. Amount of Phytosterol in PSCF

Based on the area under the curve calculations and comparison with phytosterol standards, 100 g of PSCF was found to contain 50.92 ± 1.27 mg of β-sitosterol, 21.21 ± 0.35 mg of stigmasterol, and 3.43 ± 1.13 mg of campesterol. Brassicasterol was not detected.

### 5.2. Peroxide Value

PSCF contains unsaturated fatty acids, which are susceptible to auto-oxidation at the double bonds when exposed to air. The peroxide value was measured using HPLC to evaluate the extent of oil oxidation. The analysis revealed a peroxide value of 6.41 ± 0.58 mEq peroxide/kg fat, indicating the level of oxidative stability of the PSCF.

### 5.3. Color Measurement

**Figure 2:**
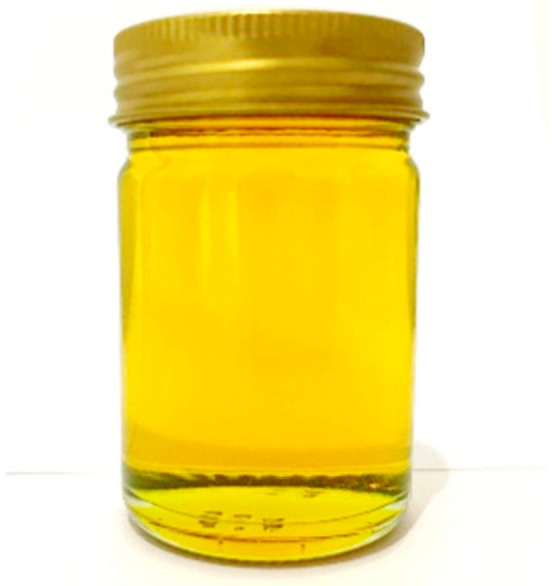
Appearance of PSCF.

As presented in Figure 2, the color analysis of the PSCF indicated an L* (brightness) value of 59.27 ± 0.07, a* (redness) value of −5.68 ± 0.06, and b* (yellowness) value of 78.49 ± 0.01. These results confirm that the PSCF exhibits a characteristic yellow color.

### 5.4. Rheology of PSCF

**Figure 3:**
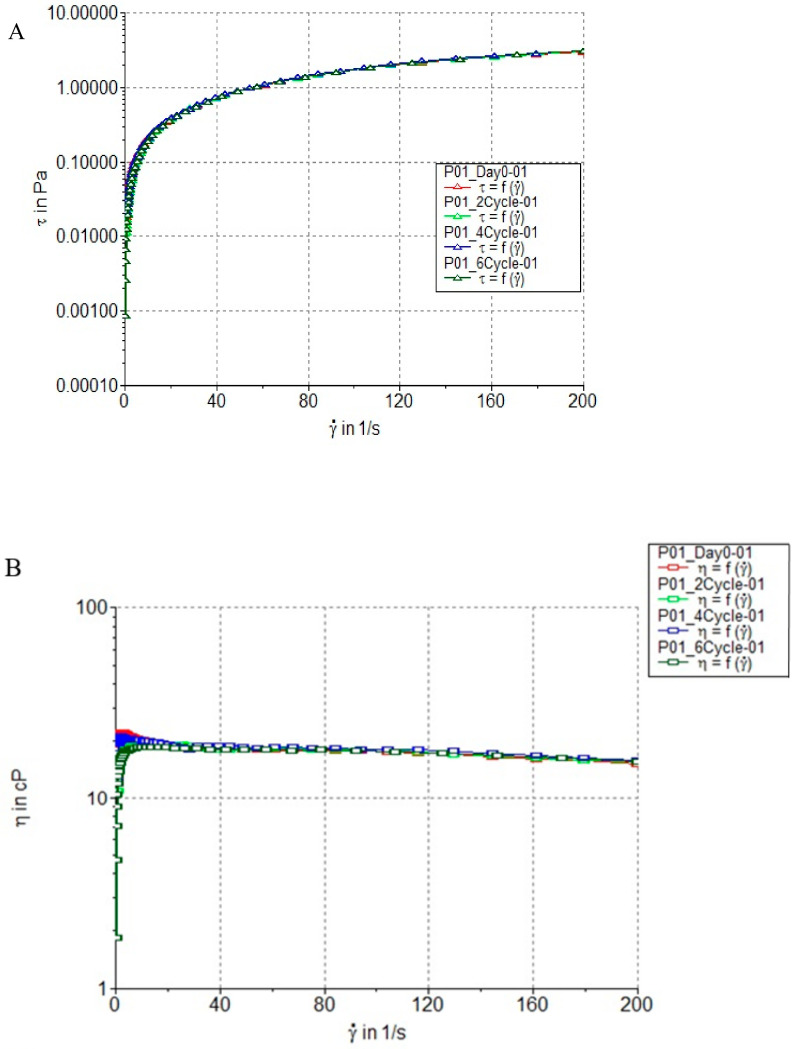
Rheology measurement. (**A**) Flow curves of PSCF expressed as shear rate and applied shear stress. (**B**) Flow curves of PSCF expressed as viscosity and shear rate.

Figure 3A shows the relationship between shear stress and shear rate. The curve indicates that PSCF exhibits pseudoplastic (shear-thinning) behavior. Initially, the shear stress increases rapidly with increasing shear rate, but at higher shear rates, the slope becomes less steep, indicating a reduction in viscosity as the shear rate increases. This behavior suggests that the spray becomes easier to spread or apply as more force is applied, which is a desirable characteristic in topical formulations for ease of application.

Figure 3B shows the viscosity of PSCF plotted against the shear rate. The viscosity decreases with increasing shear rate, confirming the pseudoplastic nature of the spray. This means that PSCF has a higher viscosity at rest, providing good adhesion to the skin, but thins out when subjected to shear forces, ensuring smooth and easy spreading on the affected area.

The results show minimal variation across multiple test cycles (Day 0, 2 cycles, 4 cycles, and 6 cycles), indicating that the rheological properties of PSCF remain stable over time and under different storage conditions. This suggests good physical stability of the formulation, ensuring consistent performance throughout its shelf life.

### 5.5. Demographic Characteristics

This randomized controlled trial included 66 participants with chronic shoulder pain lasting over three months, recruited from the Thai Traditional Medicine Clinic at Phetchaburi Rajabhat University’s Faculty of Nursing and Allied Health Sciences. This study was performed following the CONSORT guideline, which can be found in the Supplementary Materials. Out of 74 screened individuals, 66 met the eligibility criteria and were randomly assigned into two groups: PSCF (n = 33) and DS (n = 33), as detailed in Table 2. The baseline characteristics between groups were analyzed using independent *t*-test for continuous data and chi-square test for categorical data. Both groups were not statistically different in terms of age, gender, BMI, pain period, and shoulder pain patterns.

### 5.6. Pain Intensity

The baseline Visual Analog Scale (VAS) pain scores were similar between the PSCF (6.17 ± 1.11) and DS (6.16 ± 0.98) groups, with no significant difference observed. Both groups experienced significant pain reduction over the study period (*p* < 0.001). In the PSCF group, mean pain scores decreased from 6.17 ± 1.11 at baseline to 4.43 ± 0.88 at week 1 and 2.97 ± 0.83 at week 2. Similarly, the DS group showed reductions from 6.16 ± 0.98 to 4.41 ± 0.99 at week 1 and 2.81 ± 0.86 at week 2. No significant differences in pain scores were observed between the two groups at any time point, as shown in Table 3.

### 5.7. Neck Disability Index (NDI)

The neck disability scores at baseline were comparable between the PSCF group (20.20 ± 1.93) and the diclofenac spray (DS) group (20.08 ± 1.71). Both groups demonstrated significant reductions in disability scores at week 1 (PSCF: 11.55 ± 1.64; DS: 11.56 ± 1.10) and week 2 (PSCF: 8.34 ± 1.69; DS: 8.33 ± 1.43) compared to baseline (*p* < 0.001). No significant differences in neck disability scores were observed between the two groups at any time point, as detailed in Table 3.

### 5.8. Pressure Pain Threshold (PPT)

The mean pressure pain threshold (PPT) at baseline was 2.70 ± 0.59 in the PSCF group and 2.69 ± 0.63 in the DS group, with no significant difference between the groups. Following the intervention, the PSCF group exhibited significant increases in PPT at week 1 (4.81 ± 0.92) and week 2 (5.73 ± 0.57) compared to baseline (*p* < 0.001). Similarly, the DS group showed significant increases in PPT at week 1 (4.80 ± 0.81) and week 2 (5.73 ± 0.57) compared to baseline (*p* < 0.001). No significant differences in PPT were observed between the two groups at any time point during the 2-week study period, as shown in Table 3.

### 5.9. Range of Motion

Cervical range of motion (CROM) was assessed at baseline, week 1, and week 2 across six movements: flexion, extension, right lateral flexion, left lateral flexion, right cervical rotation, and left cervical rotation. The results, detailed in Table 4, indicated no significant baseline differences between the PSCF and DS groups across all movements.

Both groups significantly improved CROM (*p* < 0.001) from baseline to weeks 1 and 2. The PSCF group demonstrated improvements comparable to the DS group, with no significant differences observed between the groups at any time point. Specifically, cervical flexion increased from approximately 39° to 62°, extension from 41° to 60°, lateral flexion from 35–38° to 49–52°, and rotation from 53–55° to 71–74° in both groups by week 2, as shown in Table 4.

### 5.10. Health-Related Quality of Life

After two weeks of treatment, both the PSCF and DS groups demonstrated improvements across all eight health domains of the SF-36. The highest scores were recorded in physical functioning (PSCF: 75.61 ± 15.04; DS: 76.06 ± 13.21), followed by emotional role (PSCF: 66.67 ± 12.23; DS: 66.97 ± 15.56) and vitality (PSCF: 64.85 ± 16.18; DS: 65.45 ± 17.61). Other domains, including physical role, general health, social functioning, mental health, and bodily pain (PSCF: 54.24 ± 12.88; DS: 54.09 ± 9.31), also showed moderate improvements. No significant differences were observed between the two groups in any domain, as detailed in Table 5.

## 6. Discussion

This study aimed to investigate the chemical composition and physical stability of PSCF. The analysis revealed a saturated fatty acid profile in descending order of abundance: lauric acid > myristic acid > palmitic acid > undecanoic acid > capric acid > stearic acid > caprylic acid > arachidic acid. These findings are consistent with previous studies on the composition of cold-pressed coconut oil, which reported lauric acid (50.29%), myristic acid (18.99%), caprylic acid (8.73%), palmitic acid (7.20%), capric acid (6.08%), stearic acid (2.77%), and caproic acid (0.68%) [33]. Boateng et al. report that coconut oil comprises caprylic acid, capric acid, lauric acid, myristic acid, palmitic acid, stearic acid, oleic acid, and linoleic acid [34]. Coconut oil, administered at a dose of 4000 mg/kg, has exhibited significant anti-inflammatory and analgesic effects in animal models [35].

The PSCF contained β-sitosterol at 50.92 ± 1.27 mg/100 g. In vitro studies have demonstrated that β-sitosterol exhibits optimal anti-inflammatory activity at concentrations of 50–100 μM, primarily by inhibiting nuclear factor-kB (NF-kB) p65 phosphorylation, a crucial mechanism in inflammatory regulation [36]. Animal studies revealed that the oral administration of β-sitosterol at 200 mg/kg showed superior efficacy in reducing inflammation compared to ibuprofen (by 17%) and prednisone (by 11%). The topical application of β-sitosterol at a dose of 1.5 mg/ear produced optimal anti-inflammatory effects [37]. The intraperitoneal administration of β-sitosterol (20 mg/kg) demonstrated superior analgesic effects compared to paracetamol (50 mg/kg) in the hot plate test, effectively reducing peripheral pain and decreasing writhing responses [38].

Furthermore, the PSCF contained curcumin 28.73 ± 5.73 mg/100 g. Curcumin exerts its anti-inflammatory effects through four primary mechanisms. It inhibits key signaling pathways, including NF-κB via TLR4 and PPARγ binding, as well as MAPK, AP-1, JAK/STAT, and the NLRP3 inflammasome [39]. Additionally, curcumin reduces inflammatory mediators such as IL-1, IL-1β, IL-6, IL-8, IL-17, IL-27, TNF-α, iNOS, NO, RANTES, G-CSF, and MCP-1. It also regulates immune cell activity by modulating Th17 cells, balancing Treg/Th17 dynamics, and influencing dendritic cell function [40]. The physical analysis of PSCF revealed a peroxide value of 6.41 ± 0.58 mEq peroxide/kg fat, attributed to coconut oil processing at 180 °C for 3 h. This is consistent with Srivastava et al.’s findings, where coconut oil peroxide values increased from 3.25 mEq peroxide/kg fat initially to 9.12 mEq peroxide/kg fat after 6 h at 180 °C, before decreasing to 8.01 mEq peroxide/kg fat after 8 h, all within the acceptable threshold of 10 mEq peroxide/kg fat [41]. The formulation also demonstrated a skin-compatible pH of 5.00 ± 0.05, falling within the normal skin pH range of 4–7 [42]. The yellow color of the product, reflected in a b* value of 78.49 ± 0.01, suggested the presence of curcumin in PSCF [43].

The second objective of this study was to compare the efficacy of PSCF and DS in treating chronic shoulder muscle pain by evaluating pain intensity, range of motion, neck disability, and health status. The findings supported the research hypothesis, showing no significant differences between the PSCF and DS groups across all measured parameters. The pain-relieving effects of PSCF can be attributed to its active ingredients, particularly menthol and curcumin.

Menthol’s mechanism of action involves the activation of TRPM8 receptors, which reduces the stimulation of C nociceptor and A delta fibers, thereby interrupting pain signal transmission to the brain [44]. Additionally, menthol exhibits analgesic properties through k-opioid receptor binding [45]. This aligns with studies demonstrating significant pain reduction with 30% menthol-containing topical products in healthy volunteers with induced pain sensitivity. However, efficacy varies based on epidermal thickness, application duration, and skin surface area [46].

Curcumin contributes to pain relief through its comprehensive anti-inflammatory mechanisms, including the inhibition of inflammatory enzymes (phospholipase, lipoxygenase, cyclooxygenase-2, collagenase, elastase, hyaluronidase), inflammatory mediators (leukotrienes, thromboxane, prostaglandins, nitric oxide), and immune-related proteins such as TNF and IL-12 [47]. These combined mechanisms highlight PSCF’s therapeutic potential for managing chronic shoulder muscle pain. Animal studies have demonstrated curcumin’s analgesic and anti-inflammatory effects through the modulation of the central nervous and opioid systems, leading to increased levels of pain-relieving neurotransmitters such as dopamine, serotonin, and noradrenaline [48]. These findings align with Boonruab et al., who reported significant pain reduction in chronic shoulder muscle pain patients treated with a curcumin-containing herbal compress over two weeks [49].

Regarding pressure pain threshold (PPT), Behm et al. found that menthol-containing ointments effectively increased PPT in patients with various musculoskeletal conditions [50]. Similarly, our findings are consistent with Wisuitiprot et al.’s study, which demonstrated comparable PPT increases following six consecutive days of Phlai oil application and diclofenac gel treatment [24].

Cervical range of motion (CROM), a critical measure of muscle tension in chronic muscle pain patients with limited mobility, showed statistically significant improvements across all movements (flexion, extension, right and left lateral flexion, and right and left rotation) in both the PSCF and DS groups. These improvements correlate with findings by Sadria et al. highlighting the role of upper back muscle contraction patterns, and Yu et al., who reported reduced muscle pain with similar interventions [51,52]. These results collectively support the efficacy of PSCF in improving musculoskeletal health outcomes.

Our results align with Wisuitiprot et al.’s findings on the efficacy of Phlai oil in improving cervical range of motion and Hayeeyahya et al.’s study, which reported significant improvements in range of motion using coconut oil formulations containing Phlai, ginger, and white turmeric extracts [24,53]. Health status assessments in chronic shoulder muscle pain patients revealed that the PSCF group achieved the greatest improvement in physical mobility dimensions. At the same time, the DS group also showed notable improvements in physical mobility, but minimal changes in overall health status. Both groups demonstrated statistically significant improvements across all health status parameters. These findings correspond with Niempoog et al.’s study, which reported enhanced quality of life in osteoarthritis patients treated with diclofenac gel and Plygersic (a ginger and Phlai extract gel), as well as Boonruab et al.’s research, which found comparable quality of life improvements between Phlai-containing herbal compresses and diclofenac gel in patients with chronic shoulder muscle pain [23,54].

Additionally, research studies have reported that the comparative analysis of Thai massage therapy versus topical diclofenac for myofascial pain syndrome demonstrated superior therapeutic outcomes with massage intervention. The findings revealed that the massage therapy group exhibited more significant improvements in pain reduction (VAS scores), physical parameters (cervical range of motion, pressure pain threshold), and quality of life measures (SF-36 scores) [55]. Physical therapy interventions offer various treatment options, with thermotherapy aiding circulation and healing, ultrasound providing direct mechanical energy to trigger points, electrical therapy assisting pain control, and laser therapy delivering pain relief through mechanisms that remain unclear [56].

A review of recent studies on treatments for myofascial pain syndrome (MPS) reveals promising short-term effects from a variety of interventions. Aldemir et al. (2007) compared five treatment methods over a one-week period, including Botox-A injection, lidocaine injection, conventional ultrasound, high-power pain threshold ultrasound, and muscle stretching exercises. Assessments using the Visual Analog Scale showed significant pain reduction in all treatment groups at one-week post intervention [57]. Meanwhile, a study by Naewboot and Kanchanatawan (2016) provided 20 MPS patients with three Thai massage sessions within one week. The results demonstrated significant decreases in anxiety levels, depression scores, and pain intensity, with all changes being statistically significant. Furthermore, patients with higher initial levels of anxiety and depression exhibited greater therapeutic responses [58]. The most recent study by Boonruab et al. (2021) compared the effectiveness of Court-Type Traditional Thai Massage (CTTM) versus Thai Hermit Exercise (THE) in increasing cervical range of motion (CROM) and reducing pain from MPS in the upper trapezius muscle. Evaluations were conducted before the study began, on day 7, and on day 11 post intervention. The findings revealed that both the CTTM and THE groups experienced significantly decreased pain levels and increased cervical range of motion [32]. In summary, a diverse range of treatments, including injections, ultrasound, massage, and exercise, have demonstrated efficacy in alleviating pain and improving function in patients with myofascial pain syndrome over a short-term period of 1–2 weeks.

The study demonstrates that PSCF provides therapeutic benefits comparable to DS in managing myofascial pain syndrome of the upper trapezius. Over the two-week intervention, both PSCF and DS groups showed significant improvements in pain intensity, cervical range of motion (CROM), Neck Disability Index (NDI), and pressure pain threshold (PPT). Notably, there were no significant differences between the two groups across these clinical parameters, indicating that PSCF can be an effective alternative to DS for relieving musculoskeletal pain. The comparable efficacy is largely attributed to the active ingredients in PSCF, including curcumin, menthol, and β-sitosterol, which collectively provide anti-inflammatory and analgesic effects.

One of the key advantages of PSCF over DS is its improved safety profile. Diclofenac, a commonly used non-steroidal anti-inflammatory drug (NSAID), is known to carry dose-dependent risks of gastrointestinal, cardiovascular, and renal side effects. These systemic effects are associated with reduced prostaglandin synthesis, which is critical in maintaining gastrointestinal and renal homeostasis [59]. Although the risk is lower with topical applications than oral NSAIDs, diclofenac spray can still cause skin irritation, including erythema, dryness, and itching [29]. A study comparing oral diclofenac potassium and placebo for postoperative pain in adults found that diclofenac potassium 50 mg was most effective. A few serious adverse events were reported, indicating safety for short-term use [60]. In a comparative analysis of oral versus topical diclofenac administration among patients receiving panretinal photocoagulation treatment, oral diclofenac demonstrated a trend toward enhanced pain reduction efficacy compared to its topical counterpart. However, this difference was not statistically significant [61]. Hoang reported the side effects and tolerability of oral Phlai capsules, which were monitored throughout the study period through follow-up interviews. The most frequently reported adverse events were sedation, dizziness, dry mouth/nose, and headache [62]. In contrast, PSCF was well tolerated by participants, with no adverse skin reactions reported, making it a safer alternative for long-term use in patients with chronic musculoskeletal conditions.

Additionally, PSCF offers skin compatibility with a pH level of 5.00 ± 0.05, closely matching the natural pH range of human skin (4–7). The natural composition of PSCF, including coconut oil, enhances skin hydration and reduces the risk of irritation. Moreover, the bioactive compounds in PSCF, such as curcumin (28.73 mg/100 g), β-sitosterol (50.92 mg/100 g), and lauric acid (38.86 g/100 g), contribute to its anti-inflammatory, analgesic, and antioxidant properties. Curcumin exerts its therapeutic effects by modulating key inflammatory pathways, including NF-κB and MAPK, while β-sitosterol inhibits pro-inflammatory cytokines, further reducing pain and inflammation. Menthol, another key ingredient, activates TRPM8 receptors, providing a cooling sensation and interrupting nociceptive signal transmission, thereby enhancing pain relief.

Beyond its comparable efficacy and safety, PSCF has additional advantages due to its natural and sustainable origin. Phlai (*Zingiber montanum*) has been used for centuries in traditional Thai medicine for its anti-inflammatory and pain-relieving properties. By incorporating traditional knowledge and using locally sourced ingredients, PSCF not only supports sustainable healthcare practices, but also offers an alternative treatment with cultural significance. Furthermore, using coconut oil as a carrier in PSCF enhances its stability and bioavailability, as evidenced by its peroxide value (6.41 ± 0.58 mEq peroxide/kg fat), which remains within acceptable limits for topical formulations.

Therefore, PSCF presents a viable and safer alternative to diclofenac spray for treating MPS. It has comparable efficacy in pain reduction and functional improvement, a favorable safety profile, and additional benefits related to its natural composition and traditional origin. These findings highlight PSCF as a promising option for patients seeking effective pain relief with minimal risk of systemic side effects. Future studies with a larger sample size and multi-center trials are recommended to enhance the external validity and generalizability of the findings.

## 7. Conclusions

A novel PSCF formulation, incorporating coconut oil-extracted compounds, β-sito- sterol, and curcumin, demonstrated robust physicochemical stability. Clinical assessment following one and two weeks of application in chronic shoulder muscle pain patients showed significant improvements across multiple parameters, including pain intensity, pressure pain threshold, mobility, and health status measures, without adverse reactions. The therapeutic outcomes were comparable to those from diclofenac spray, establishing this formulation as a viable topical analgesic alternative for muscle pain management with one week of continuous application.

## Figures and Tables

**Table 1:** Fatty acid composition of PSCF.

Fatty Acid	Amount (g/100 g of PSCF)
Saturated fatty acid	
Lauric acid (C12:0)	38.86 ± 1.72
Myristic acid (C14:0)	17.52 ± 0.35
Palmitic acid (C16:0)	9.88 ± 2.01
Undecanoic acid (C11:0)	8.76 ± 0.50
Capric acid (C10:0)	3.72 ± 1.28
Stearic acid (C18:0)	2.94 ± 1.15
Caprylic acid (C 8:0)	2.30 ± 0.30
Arachidic acid (C 20:0)	0.09 ± 0.83
Unsaturated fatty acid	
Cis-9-Oleic acid (C18:1n9c)	7.87 ± 1.05
Linoleic acid (C18:2n6c)	1.90 ± 0.81

**Table 2:** Demographic characteristics of participants.

Characteristics	Participant Groups		*p*-Value
	PSCF (n = 33)	Diclofenac Spray (n = 33)	
Age (years) ^a^	30.39 ± 12.24	30.36 ± 12.69	0.992
Gender ^b^			1.000
Female	20 (60.60%)	21 (63.60%)	
Male	13 (39.40%)	12 (36.40%)	
BMI ^a^	22.55 ± 3.11	21.52 ± 4.13	0.254
Pain period (months) ^a^	5.58 ± 0.66	5.58 ± 0.49	0.949
Shoulder pain patterns ^b^			0.958
Right	8 (24.20%)	8 (24.20%)	
Left	9 (27.30%)	10 (30.30%)	
Both sides	16 (48.50%)	15 (45.50%)	

Abbreviations: PSCF, Phlai (*Zingiber montanum*) spray cool formula; DS, diclofenac spray. ^a^ Mean ± standard deviation. ^b^ Number (percentage).

**Table 3:** VAS, NDI, and PPT parameters within and between groups measured at baseline, week 1, and week 2.

Parameter	Baseline (Mean ± SD)	Week 1 (Mean ± SD)	Week 2 (Mean ± SD)	F *(p*-Value ^b^)	ηp^2^	Score Changes Between Baseline and Week 2	
						Mean ± SD	95% CI
VAS							
PSCF (n = 33)	6.17 ± 1.11	4.43 ± 0.88 ***	2.97 ± 0.83 ***	197.13 *(p* < 0.001)	0.860	3.20 ± 1.15	2.79–3.61
DS (n = 33)	6.16 ± 0.98	4.41 ± 0.99 ***	2.81 ± 0.86 ***	213.09 *(p* < 0.001)	0.869	3.35 ± 1.07	2.97–3.73
*p*-value ^a^	0.969	0.931	0.439				
NDI							
PSCF (n = 33)	20.20 ± 1.93	11.55 ± 1.64 ***	8.34 ± 1.69 ***	1523.27 *(p* < 0.001)	0.979	11.85 ± 1.39	11.36–12.35
DS (n = 33)	20.08 ± 1.71	11.56 ± 1.10 ***	8.33 ± 1.43 ***	871.26 *(p* < 0.001)	0.965	11.74 ± 1.50	11.22–12.28
*p*-value ^a^	0.788	0.977	0.979				
PPT							
PSCF (n = 33)	2.70 ± 0.59	4.81 ± 0.92 ***	5.73 ± 0.57 ***	160.76 *(p* < 0.001)	0.834	3.03 ± 0.75	2.76–3.30
DS (n = 33)	2.69 ± 0.63	4.80 ± 0.81 ***	5.72 ± 0.68 ***	175.63 *(p* < 0.001)	0.846	3.03 ± 0.83	2.74–3.33
*p*-value ^a^	0.947	0.957	0.948				

ηp^2^: partial eta squared. ^a^ *p*-value calculated by independent sample *t*-test for between-group comparison. ^b^ *p*-value calculated by repeated-measures ANOVA to determine differences within groups in different periods. *** indicates *p* < 0.001.

**Table 4:** Cervical range of motion (CROM) parameters within and between groups, measured at baseline, week 1, and week 2.

CROM	Baseline (Mean ± SD)	Week 1 (Mean ± SD)	Week 2 (Mean ± SD)	F *(p*-Value ^b^)	ηp^2^	Score Changes Between Baseline and Week 2	
						Mean ± SD	95% CI
Flexion							
PSCF	39.01 ± 2.35	50.27 ± 4.14 ***	62.56 ± 3.21 ***	444.75 *(p* < 0.001)	0.933	23.54 ± 4.09	22.09–25.10
DS	39.07 ± 2.53	50.26 ± 3.58 ***	62.57 ± 4.22 ***	351.56 *(p* < 0.001)	0.917	23.49 ± 5.04	21.71–25.28
*p*-value ^a^	0.920	0.992	0.991				
Extension							
PSCF	41.22 ± 2.01	56.11 ± 4.29 ***	60.66 ± 2.94 ***	352.91 *(p* < 0.001)	0.917	19.43 ± 3.20	18.30–20.57
DS	41.21 ± 1.73	56.14 ± 3.19 ***	60.65 ± 2.34 ***	587.80 *(p* < 0.001)	0.948	19.43 ± 2.92	18.40–20.47
*p*-value ^a^	0.983	0.974	0.988				
Right lateral flexion							
PSCF	38.40 ± 3.40	44.89 ± 4.00 ***	49.03 ± 4.23 ***	65.28 *(p* < 0.001)	0.671	10.63 ± 5.14	8.81–12.46
DS	38.38 ± 3.33	44.90 ± 4.13 ***	49.00 ± 3.20 ***	78.74 *(p* < 0.001)	0.711	10.61 ± 4.52	9.01–12.22
*p*-value ^a^	0.990	0.992	0.974				
Left lateral flexion							
PSCF	35. 95 ± 4.34	45.45 ± 4.29 ***	52.93 ± 3.13 ***	152.58 *(p* < 0.001)	0.827	16.97 ± 5.79	14.92–19.04
DS	35.92 ± 4.08	45.44 ± 3.84 ***	52.92 ± 3.10 ***	206.13 *(p* < 0.001)	0.866	17.00 ± 4.99	15.30–18.77
*p*-value ^a^	0.977	0.992	0.990				
Right cervical rotation							
PSCF	55.51 ± 3.26	63.36 ± 3.80 ***	71.14 ± 3.38 ***	136.19 *(p* < 0.001)	0.810	15.63 ± 5.18	13.80–17.48
DS	55.52 ± 2.93	63.35 ± 2.83 ***	71.11 ± 1.97 ***	274.20 *(p* < 0.001)	0.895	15.59 ± 3.60	14.32–16.87
*p*-value ^a^	0.989	0.990	0.965				
Left cervical rotation							
PSCF	53.78 ± 2.60	67.00 ± 2.26 ***	74.12 ± 2.79 ***	494.45 *(p* < 0.001)	0.939	20.34 ± 3.96	18.94–21.75
DS	53.77 ± 3.37	67.03 ± 2.87 ***	74.11 ± 2.57 ***	495.78 *(p* < 0.001)	0.939	20.34 ± 4.02	18.92–21.77
*p*-value ^a^	0.989	0.962	0.988				

^a^ *p*-value calculated by independent sample *t*-test for between-group comparison. ^b^ *p*-value calculated by repeated-measures ANOVA to determine differences within groups in different periods. *** indicates *p* < 0.001.

**Table 5:** The 36-Item Short-Form Health Survey (SF-36) outcomes at baseline and week 2 including within- and between-group differences.

36-Item Short-Form Health Survey	PSCF (n = 33)	DS (n = 33)	*p*-Value ^b^
Overall Health Status			
Baseline	55.30 ± 10.82	56.97 ± 12.80	0.570
Week 2	63.94 ± 9.42 ***	64.70 ± 13.11 *	0.788
*p*-value ^a^	<0.001	0.024	
Physical Functioning			
Baseline	69.24 ± 14.90	66.06 ± 12.42	0.350
Week 2	75.61 ± 15.04 ***	76.06 ± 13.21 ***	0.897
*p*-value ^a^	<0.001	0.001	
Social Function			
Baseline	54.70 ± 11.38	55.00 ± 14.25	0.924
Week 2	63.18 ± 9.75 ***	63.33 ± 9.16 *	0.948
*p*-value ^a^	<0.001	0.011	
Physical Role Function			
Baseline	41.12 ± 19.06	42.58 ± 10.83	0.705
Week 2	64.55 ± 12.46 ***	65.45 ± 9.55 ***	0.740
*p*-value ^a^	<0.001	<0.001	
Emotional Role Function			
Baseline	58.33 ± 14.83	58.21 ± 17.20	0.976
Week 2	66.67 ± 12.23 ***	66.97 ± 15.56 **	0.930
*p*-value ^a^	<0.001	0.006	
Pain			
Baseline	42.12 ± 11.46	41.52 ± 7.23	0.798
Week 2	54.24 ± 12.88 ***	54.09 ± 9.31 ***	0.956
*p*-value ^a^	<0.001	<0.001	
Energy			
Baseline	52.58 ± 13.87	53.49 ± 10.86	0.768
Week 2	64.85 ± 16.18 ***	65.45 ± 17.61 **	0.885
*p*-value ^a^	<0.001	0.001	
Mental Health			
Baseline	53.79 ± 12.31	54.55 ± 13.25	0.811
Week 2	62.58 ± 12.98 ***	63.48 ± 9.23 **	0.744
*p*-value ^a^	<0.001	0.003	

^a^ *p*-value calculated by paired *t*-test for within-group comparison. ^b^ *p*-value calculated by independent-sample *t*-test for between-group comparison. * indicates *p* < 0.05, ** indicates *p* < 0.01, and *** indicates *p* < 0.001.

## Data Availability

The original contributions presented in this study are included in the article/Supplementary Material. Further inquiries can be directed to the corresponding author(s).

## Supplementary Materials

The following supporting information can be downloaded at: https://www.mdpi.com/article/10.3390/life15030360/s1, This study was performed following the CONSORT guideline, which can be found in the Supplementary Materials.
